# In Vitro Effect of Estradiol and Progesterone on Ovine Amniotic Epithelial Cells

**DOI:** 10.1155/2019/8034578

**Published:** 2019-03-27

**Authors:** Annunziata Mauro, Hashimita Sanyal, Angelo Canciello, Paolo Berardinelli, Valentina Russo, Nicola Bernabò, Luca Valbonetti, Barbara Barboni

**Affiliations:** Faculty of Bioscience and Technology for Food, Agriculture and Environment, University of Teramo, Via R. Balzarini 1, 64100 Teramo, Italy

## Abstract

Amniotic epithelial cells (AECs), an emerging source of extrafoetal stem cells, have recently attracted attention for their great regenerative potential. Since AEC amplifications are accompanied by the loss of their native epithelial phenotype and by the progressive reduction of relevant biological properties, the issue to be addressed is the development of effective culture protocols. In this context, recently, it has been demonstrated that progesterone (P_4_) supplementation during ovine AEC (oAEC) expansion could prevent the undesirable epithelial-mesenchymal transition (EMT). In contrast, there is no information to date on the role of the other pregnancy steroids in culture. With this aim, the present study has been designed to clarify the impact of estradiol (E_2_), alone or in combination with P_4_ (12.5 *μ*M and 25 *μ*M), during oAEC amplification. Steroid supplementations were assessed by testing oAEC proliferation, stemness, EMT, and osteogenic or chondrogenic plasticity. The results indicated that EMT can be prevented exclusively in the presence of high doses of P_4_, while it occurred rapidly in cells exposed to E_2_ as denoted by protein (cytokeratin-8 and alpha-SMA) and gene expression (*vimentin* and *snail*) profiles. Moreover, steroid exposure was able to influence highly oAEC plasticity. Particularly, P_4_-treated cells displayed a precommitment towards osteogenic lineage, confirmed by the upregulation of *OCN*, *RUNX2*, and the greater deposition of calcium nodules. Conversely, P_4_ exposure inhibited oAEC chondrogenic differentiation, which was induced in E_2_-treated cells as confirmed by the upregulation of chondrogenesis-related genes (*SOX9*, *ACAN*, and *COL2A1*) and by the accumulation of Alcian blue-positive extracellular matrix. Simultaneously, E_2_-treated cells remained unresponsive to osteogenic inductive stimuli. In conclusion, media supplementation with high doses of steroids may be adopted to modulate phenotype and plasticity during oAEC amplification. Relevantly, the osteo or chondro steroid-induced precommitment may open unprecedented cell-based therapies to face the unsolved orthopaedic issues related to osteochondral regeneration.

## 1. Introduction

Stem cell-based regenerative medicine represents one of the most relevant challenges of the modern biomedical sciences. In this context, amniotic-derived epithelial cells (AECs) have assumed a relevant role due to their promising regenerative attitude [1–9].

By virtue of their early embryonic origin, this extrafoetal source of stem cells expresses, in a highly conserved manner [10–12], several embryonic markers, such as SSEA-3, SSEA-4, TRA-1-60, and TRA-1-81 and pluripotent genes (*OCT4*, *SOX2*, *NANOG*, and *TERT*), probably involved in driving their great differentiation potential [11–14]. A relevant biological advantage of this stem cell source is their low immunogenicity [14, 15] that, combined with their innate immunomodulatory and anti-inflammatory activities [14, 15], have allowed their safe use as immunocompetent individuals under allogenic and xenogenic transplantation preclinical settings [6, 11, 15–21] and clinical trials [22, 23].

Similarly, like other stem cell sources, in order to optimize the regenerative medicinal use of AECs, one technical issue has to be considered: the standardization of the *in vitro* amplification protocols, leading to the increase of the number of cells without affecting stem cell native biological properties. Currently, a validated protocol has been proposed for human AEC (hAEC) [24] even if some evidences demonstrated that it does not guarantee the persistence of the epithelial phenotype during amplification [25–28]. Indeed, the *in vitro* amplification of both hAEC and oAEC induced the spontaneous loss of the epithelial phenotype as a consequence of the epithelial-mesenchymal transition (EMT) process that occurs in culture under the influence of released paracrine/autocrine growth factors [14, 28–30]. The EMT consists of a transdifferentiation process whereby epithelial cells acquire a mesenchymal phenotype assuming wider migratory and invasive properties. EMT is a complex biological process that plays a crucial role in development, in wound healing, and in stem cell differentiation, as well as, under pathological conditions, in sustaining organ fibrosis and cancer progression [31]. Apart from the EMT physiological and pathological roles, recent evidences demonstrated that even under *in vitro* conditions, it might be responsible in changing cell functions [14, 26, 29, 30]. Our group has recently associated EMT with the progressive reduction in oAEC anti-inflammatory cytokine releasing [30] by linking this undesirable event to the inability to reproduce *in vitro* the hormonal context that modulates amniotic cell homeostasis during pregnancy. In particular, the attention was focused on progesterone (P_4_), the key steroid that sustains the whole pregnancy lifetime and that during amplification was able to preserve the cell native epithelial phenotype [30] by avoiding the reduction of the basal and induced immunomodulatory AEC activities [30]. The intracellular mechanism involved in mediating the inhibitory EMT role of P_4_ was related to its interference with the TGF-*β* autocrine/paracrine signaling pathway [14, 28–30], thus increasing the evidences of P_4_ modulatory role on the amniotic membrane [32–35]. However, while the influence of P_4_ in preserving epithelial phenotype during expansion can be considered an established evidence [30, 36–39], the influence of other pregnancy steroids remains to be assessed [32]. Therefore, since the stem cell amplification represents the first critical technological step to standardize regenerative medicine protocols, before moving them towards clinical applications, the present research has been designed to assess the effects of both estradiol (E_2_) and P_4_, during the process of amplification. With this aim, high doses of steroid supplementations (12.5 *μ*M and 25 *μ*M), alone or in combination, were added during oAEC amplification and their impact on proliferation, stemness, phenotype, EMT, and osteogenic/chondrogenic plasticity was assessed.

## 2. Materials and Methods

### 2.1. Ethic Statement

Nonethic statement was required for this research since the amniotic membranes were collected from animals of the local slaughter houses.

### 2.2. oAEC Isolation, Treatment, and Culture

**Figure 1:**
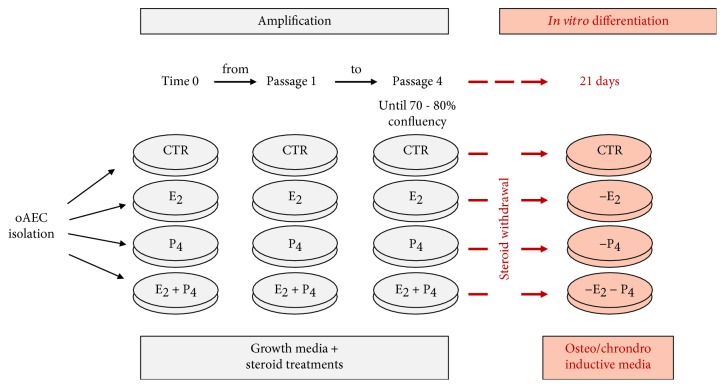
Schematic representation of the experimental design. Under the grey banner, the amplification protocols are briefly summarized: starting from freshly isolated oAEC, cells were cultured in growth medium in the absence (CTR) or in presence of different steroid treatments: E_2_ and P_4_ alone or in combination (E_2_+P_4_) at two concentrations (12.5 *μ*M or 25 *μ*M). This incubation, aimed at promoting cell expansion, was carried out until passage 4. Under the pink banner is the summarized differentiation procedures: at passage 4, once oAEC reached 70-80% of confluency, steroids were withdrawn and the cells were incubated for 21 days under differentiation conditions by exposing them to osteo- or chondrodifferentiation medium. oAECs: ovine amniotic epithelial cells; CTR: control cell; E_2_: estradiol; P_4_: progesterone.

The sheep uteri were collected at a local abattoir from sheep of Appenninica breed (*n* = 3 animals) at mid gestational stage determined on the basis of fetus dimension (ranging from 20 to 30 cm length) and brought at approximately 25°C to the laboratory in maximum 1 h, for further processing. Cell extraction was performed as previously described [30] in order to obtain membrane pieces of approximately 3–5 cm. Membrane pieces, after washed in phosphate-buffered saline (PBS), were incubated twice in 0.25% Trypsin-EDTA 200 mg/l at 37.5°C for 20 min and 30 min. The cell suspension obtained after an enzymatic digestion was filtered through a 40 *μ*m filter, and isolated cells were collected into a tube containing 10% fetal calf serum (FCS, Lonza) in order to inactivate trypsin. Freshly isolated oAECs were seeded in Petri dishes (Corning) at the final concentration of 20,000 cells/ml in alpha Eagle's minimum essential medium (*α*-MEM, Gibco) supplemented with 20% FCS, 1% UltraGlutamine (Lonza), 100 U/ml penicillin (Lonza), 100 *μ*g/ml streptomycin (Lonza), and 2.5 *μ*g/ml amphotericin (EuroClone). The cells were incubated at 38.5°C in 5% CO_2_ in the absence (CTR) or in presence of steroids E_2_ (Sigma) and/or P_4_ (Sigma) at concentrations of 12.5 *μ*M and 25 *μ*M following the experimental plan described in Figure 1. The culture media was replaced every 3 days. At 70–80% confluence, cells were dissociated by 0.05% Trypsin-EDTA and plated at the same concentration for subsequent passages till passage 3 (see Figure 1).

### 2.3. Cell Proliferation Assay

Proliferative activity of AEC cultured under CTR and steroid conditions was analyzed by MTT assay (M5655-1G, Sigma) as previously described [30]. Briefly, CTR and 12.5 and 25 *μ*M steroid-treated oAECs were seeded into 96-well plates (0.3 × 10^5^ cells/well) until reaching 70% confluence. The blank points were identified by the wells containing only culture medium (supplemented or not with steroids). Afterwards, 20 *μ*l of MTT (5 mg/ml in PBS) was added in each well and the plates were incubated at 37°C for 3.5 h. The formazan crystals were then dissolved in 100 *μ*l of DMSO. The absorbance (Abs) of the solution was measured at 595 nm and for each sample was subtracted the relative blank absorbance. The percentage (%) of proliferation was calculated as the absorbance of steroid-treated cells divided for the absorbance of CTR cells and multiplied by 100. The net absorbance of CTR cells was taken as 100% proliferation.

### 2.4. Immunohistochemistry (IHC)

Ovine AECs were evaluated for an epithelial (cytokeratin-8) and a mesenchymal protein marker (*α*-SMA) by immunofluorescence analysis according to our previous report [30]. With this aim, the cells were cultured in the presence or absence of P_4_ and E_2_, alone or in combination, on glass coverslips. Afterwards, oAECs were fixed in 4% paraformaldehyde for 10 min, incubated with 5% (*w*/*v*) BSA in PBS for 1 h at room temperature (RT), and then incubated with anti-cytokeratin-8 (1 : 200) (clone C-43, Abcam) and anti-*α*-SMA (1 : 200) (clone 1A4, Abcam) antibodies, diluted in 1% (*w*/*v*) BSA/PBS, overnight at 4°C. The immunocytochemical negative controls were performed by omitting the primary antibody and in the presence of isotype control-matched mouse IgG1 (NCG01, Abcam) for cytokeratin-8 and mouse IgG2a (Abcam) for *α*-SMA, respectively. Cy3- and Alexa Fluor 488-conjugated anti-mouse secondary antibodies, diluted 1 : 200 in 1% (*w*/*v*) BSA/PBS, were used for antigen retrieval. Nuclear counterstaining was obtained with 4′,6-diamidino-2-phenylindole (DAPI, VECTASTAIN) at the final dilution of 1 : 5000 in PBS. Coverslips were finally mounted with Fluoromount (Sigma Chemical Co.) and cell samples were analysed by Nikon A1r confocal microscope interfaced to a computer workstation, provided with NIS-Elements 4.4 software (for images acquisition) and with NIS-Elements Advanced Research imaging software (for postprocessing analysis). All digital images were acquired at 400x of magnification. Thresholds for all channel (DAPI: low 198 nm, high 3138 nm, separate: 3x; FITC: low 396 nm, high 3848 nm, separate: 1x; TRIC: low 345 nm, high 3550 nm, separate: 1x; and fill holes: ON) were set and remained constant for all image acquisitions and quantifications. The software converted the images automatically to binary images in which cells (objects) were counted based on parameter restrictions (i.e., circularity and diameter). An object catalogue for each image was generated, and nonconformed parameter objects were excluded. Values of object for each image were exported in Microsoft Excel and used for statistical analyses. For each immunofluorescence reaction, animal samples (*n* = 3) were performed in triplicate. At least 100 cells for each replicate (3/animal) sample were counted in order to quantify the incidence of cytokeratin-8- and *α*-SMA-positive cells. The values were statistically analysed by GraphPad Prism 6, and the results were expressed as percentage (%) of number of positive cells.

### 2.5. Real-Time qPCR

Real-time qPCR was performed in order to compare the mRNA expression of EMT-, bone-, and cartilage-related genes (see Table 1) in cells incubated under CTR, steroids, and differentiation conditions. Freshly isolated AECs (AEC T0), bone, and cartilage isolated from ovine tissues were used as internal control for gene expression. Total mRNA was extracted by using TRIzol (Sigma) according to the manufacturer instructions. Integrity and size distribution were evaluated by 1% agarose gel electrophoresis and GelRed staining (Bioline). Quantification of total mRNA samples was assessed by using Thermo Scientific NanoDrop 2000c UV-Vis spectrophotometer at 260 nm. Digestion of genomic DNA was carried out by DNaseI (Sigma) exposing the samples for 15 minutes at RT. cDNA that was synthetized from 1 *μ*g of total RNA of each sample was used for reverse transcription reaction with Random Hexamers primer and Tetro Reverse Transcriptase (Bioline) at final volume of 20 *μ*l, according to the manufacturer instructions. Real-time qPCR analysis was performed by using SensiFAST™ SYBR Lo-ROX kit (Bioline) according to the manufacturer's instructions. The reaction was to carry out with 7500 Fast Real-time PCR System (Life Technologies) by using the two-step cycling protocol for 40 cycles (10 seconds at 95°C for denaturation and 30 seconds at 60°C for annealing/extension) followed by melt profile analysis (7500 Software v2.3). PCR efficiency of target genes and reference internal control GAPDH were evaluated by a 1 : 10 serial dilution standard curve containing 5 points of cDNA (starting from undiluted cDNA up to 100 ng), each performed in duplicate, followed by amplification using primer pair to each gene. The slope of the line was determined by plotting the Ct (*y*-axis) versus log cDNA dilution (*x*-axis) for each gene primer pair. PCR efficiency was calculated by the formula as follows: 10^(-1/slope value)^. For each gene analyzed, each sample was performed in triplicate, and values were normalized to endogenous reference gene GAPDH. The relative expression of different amplicons was calculated by the comparative Ct (ΔΔCt) method and converted to relative expression ratio (2^-ΔΔCt^) [40]. For primer details, see Table 1.

### 2.6. Osteogenic Differentiation Culture

The steroid effect on oAECs' mesenchymal lineage plasticity was tested. Steroid treatments were maintained for 4 passages until the cells reached 70-80% of confluency. Then, the steroids were withdrawn and cells were exposed to osteogenic differentiation medium (DM) (see Figure 1). The osteogenic DM consisted of *α*-MEM supplemented with 50 *μ*M ascorbic acid (Sigma), 10 mM *β*-glycerophosphate (Sigma), 0.2 *μ*M dexamethasone (Sigma), 10% FCS, 1% UltraGlutamine (10.000 UI/ml), and 1% penicillin/streptomycin as previously reported [11]. The DM was replaced every 2 days. Osteogenesis was assessed before and after 21 days in DM by evaluating the expression of Runt-related transcription factor 2 *(RUNX2*) and osteocalcin (*OCN*) bone-related gene (see Table 1) and deposition of calcium-mineralized nodules by Alizarin Red S staining [11, 17]. The staining of calcium mineral deposits was recorded using bright light microscopy.

### 2.7. Chondrogenic Differentiation Culture

Steroid treatment was maintained until cells reached 70-80% of confluency at passage 4. Then, the steroid was withdrawn and cells were exposed to chondrogenic differentiation medium (DM) (see Figure 1). In detail, chondrogenic DM comprised of *α*-MEM supplemented with 10% FBS, 10% ITS Premix (Sigma), 10^−7^ M dexamethasone, 1 *μ*M ascorbic acid, 1% sodium pyruvate (Sigma), 10 ng/ml TGF-*β*1 (Sigma), 1% UltraGlutamine, 1% penicillin/streptomycin, and 2.5 *μ*g/ml amphotericin, for 21 days [41]. The DM was replaced every 3 days. Chondrogenesis was assessed before and after 21 days in DM by evaluating the expression of chondrogenesis-related genes, SRY-related high-mobility group box 9 (*SOX9*), aggrecan XI (*ACAN*), and type II collagen (*COL2A1*) (see Table 1). In addition, the extracellular deposition of cartilage matrix was detected by Alcian blue staining [14, 42, 43]. The staining was recorded using bright light microscopy.

### 2.8. Statistical Analysis

All investigations of the experimental design were performed on each animal sample (*n* = 3 animal), and interassay variation was calculated on three different replicates. The data are expressed as mean ± SEM values obtained from the three replicate/animal samples. Statistical analysis was performed using Prism 6 (GraphPad). Two-way ANOVA was performed on data sets for two independent variables (stemness and EMT-related gene expression in CTR and steroid-treated cells over passages). One-way ANOVA with Tukey correction was adopted for multiple comparisons and performed on data sets with a single independent variable. At least a *p* value ≤ 0.05 was considered statistically significant.

## 3. Results

### 3.1. Steroid Treatments Affect oAEC Proliferation and Modulated Stemness Gene Profile

**Figure 2:**
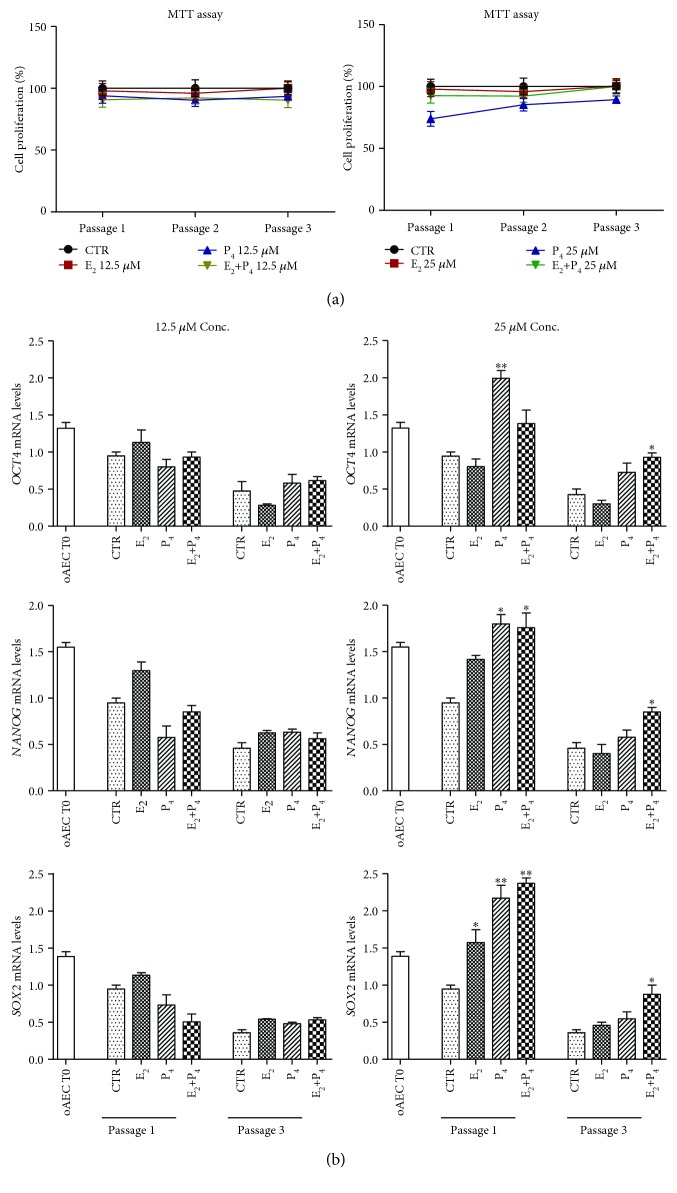
Effect of steroids on doubling time and stemness gene expression in oAECs. (a) Proliferation activity of CTR and steroid-treated oAEC (E_2_, P_4_, and E_2_+P_4_) at 12.5 *μ*M or 25 *μ*M during in vitro amplification. The data are expressed as percentage of proliferation ± SEM from values of triplicate samples obtained by three different animals, CTR set to 100%. (b) Real-time qPCR analysis of stemness gene expression profile (*OCT4*, *SOX2*, and *NANOG*) in CTR and steroid-treated oAEC at passage 1 and passage 3. Freshly isolated oAECs (AEC T0) were used as the internal control of stemness gene expression. Relative quantification of each mRNA gene expression was calculated using the ΔΔCt method and presented as fold change in gene expression normalized to endogenous *GAPDH* (internal control) and relative to the CTR at passage 1 (calibrator). Data was expressed as mean ± SEM values of samples, each performed in triplicate, obtained at least three different animals. Values were considered statistically significant for ^∗^*p* < 0.05 and ^∗∗^*p* < 0.01 with respect to the CTR values within the same passage of culture. oAECs: ovine amniotic epithelial cells; T0: time zero; CTR: control cell; E_2_: estradiol; P_4_: progesterone.

Steroid E_2_ supplementation did not affect proliferation during amplification, independently of the P_4_ presence (E_2_+P_4_) (see Figure 2(a)). On the contrary, P_4_ alone at high doses (25 *μ*M) was able to reduce cell proliferation by displaying an effect of approximately 30% lower than CTR during passage 1 and around 15% at passage 3 (see Figure 2(a)).

Moreover, steroid supplementation affected stemness gene expression in a steroid- and dose-dependent manner (see Figure 2(b)). Cell exposure to high dose of P_4_ (25 *μ*M) during the first passage stimulated an upregulation of all stemness genes (*OCT4*, *p* < 0.05; *SOX2*, *p* < 0.05; and *NANOG*, *p* < 0.05 vs. CTR cells), whereas the supplementation of high dose of E_2_ (25 *μ*M) increased exclusively the expression of *SOX2* (*p* < 0.05 vs. CTR) (see Figure 2(b)). Differently, cells exposed to high doses of E_2_ in combination with P_4_ (E_2_+P_4_ 25 *μ*M) displayed a long-term upregulation for *SOX2* and *NANOG* interested passages 1 and 3 (*p* < 0.05 vs. CTR either at passage 1 or 3) and for *OCT4* exclusive passage 3 (*p* < 0.05, see Figure 2(b)).

### 3.2. Steroid Treatments Modulate Differently the oAEC Phenotype during Amplification

**Figure 3:**
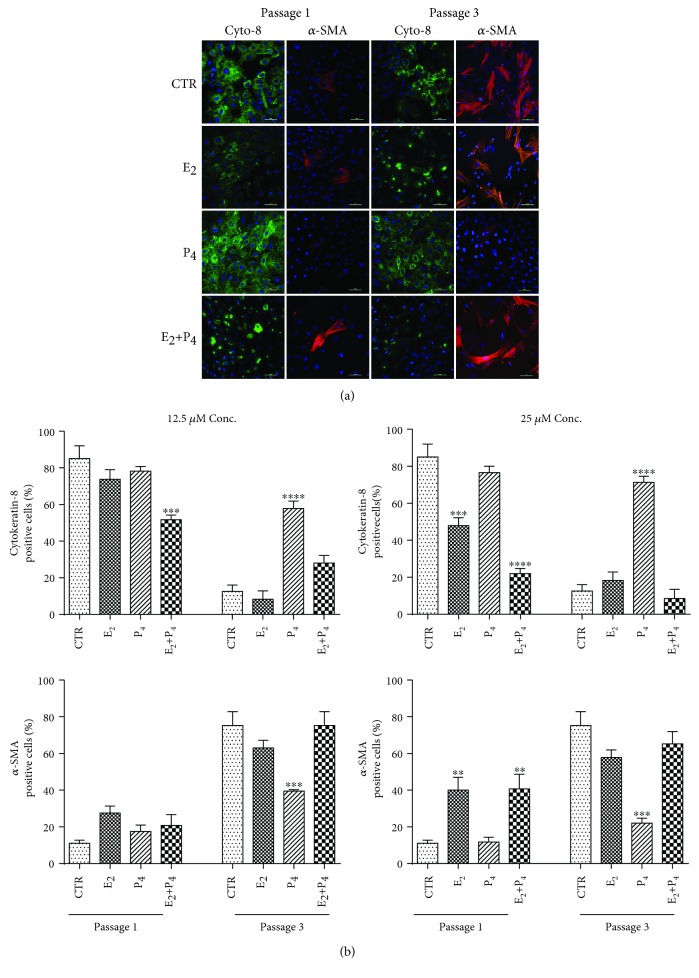
EMT steroid modulation on amplified oAEC. (a) Immunostaining for cytokeratin-8 (green) and *α*-SMA (red), epithelial and mesenchyme markers, respectively, was performed on CTR and steroid-amplified oAEC (E_2_, P4, and E_2_+P4 at 25 *μ*M), thus demonstrating that both protein profiles changed in a steroid- and passage-dependent manner (passage 1 and passage 3). Nuclei are counterstained with DAPI (blue). Scale bar: 50 *μ*m. (b) Fluorescence quantification of cytokeratin-8- and *α*-SMA-positive cells recorded in CTR and steroid-treated oAEC. This analysis was performed on cells amplified with different combinations and concentrations (12.5 *μ*M and 25 *μ*M) of steroids from passage 1 to passage 3. The data are expressed as mean ± SEM values of samples, performed in triplicate, obtained at least three different animals. Values statistically different for ^∗^*p* < 0.05, ^∗∗^*p* < 0.01, and ^∗∗∗^*p* < 0.001 in comparison to CTR within each passage. CTR: control cell; E_2_: estradiol; P4: progesterone; Cyto-8: cytokeratin-8; *α*-SMA: alpha-smooth muscle actin.

The phenotype during the *in vitro* expansion was assessed by evaluating the morphology and the incidence for the epithelial and mesenchymal markers, cytokeratin-8 and *α*-SMA, respectively (see Figure 3(a)). Untreated cells (CTR) in culture exhibited a typical epithelial phenotype confirmed by the cobblestone-like morphology and by the high positivity for cytokeratin-8 (see Figure 3(a)). Morphology, cytokeratin-8, and *α*-SMA profiles confirmed that oAECs progressively lost the native phenotype during the *in vitro* amplification carried out under CTR conditions (see Figure 3(a)) except for the cells exposed to high doses (25 *μ*M) of P_4_. This latter condition (P_4_ 25 *μ*M) was the unique cultural condition compatible with the persistence of epithelial phenotype during amplification (see Figure 3(a), P_4_) documented by the large positivity for cytokeratin-8 (*p* < 0.001 for both 25 *μ*M and 12.5 *μ*M P_4_ vs. CTR) and a low detectability for *α*-SMA (*p* < 0.001 vs. CTR) (see Figure 3(b)). On the contrary, high doses of E_2_ (25 *μ*M), added alone (E_2_) or in combination with P_4_ (E_2_+P_4_) (see Figure 3(a) and Figure 3(b)), accelerated the mesenchymal *in vitro* morphological shift that was already detectable at passage 1 when a significantly higher expression of *α*-SMA was recorded (see Figure 3(a) and Figure 3(b), *p* < 0.001 vs. CTR). At passage 3, E_2_- and E_2_+P_4_-treated cells displayed a similar phenotype (cytokeratin-8: *p* > 0.05 vs. CTR and *α*-SMA: *p* > 0.05 vs. CTR) (see Figure 3(b)).

### 3.3. EMT-Related Gene Expressions Were Differently Modulated by Steroids in oAEC

**Figure 4:**
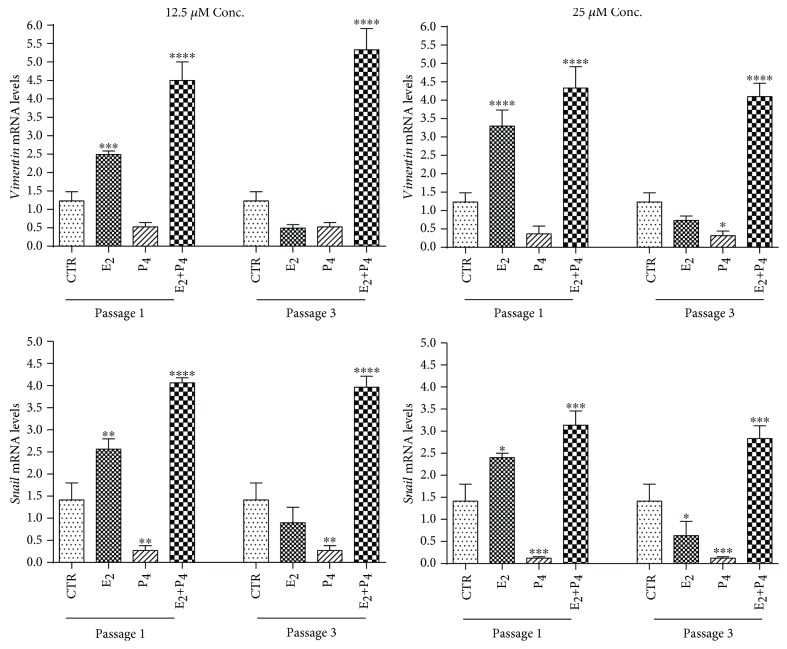
Influence of steroids on EMT gene expression during oAEC amplification. Real-time qPCR analysis of two EMT-related gene expressions (*vimentin* and *snail*) was carried out on oAEC amplified under CTR conditions or exposed to steroids at 12.5 *μ*M or 25 *μ*M (E_2_, P_4_, and E_2_+P_4_) up to passage 3. Relative quantification of each mRNA gene expression was calculated using the ΔΔCt method and presented as fold change in gene expression normalized to endogenous GAPDH (internal control) and relative to the CTR at passage 1 (calibrator). Data was expressed as mean ± SEM values of samples, each performed in triplicate, obtained at least three different animals. Values statistically different for ^∗^*p* < 0.05, ^∗∗^*p* < 0.01, ^∗∗∗^*p* < 0.001, and ^∗∗∗^*p* < 0.0001. CTR: control cell; E_2_: estradiol; P_4_: progesterone.

The role of steroids in modulating the *in vitro* EMT was better investigated by the expression of EMT-related genes, *snail* and *vimentin* (see Figure 4). Gene expression demonstrated that P_4_ independent of the doses was able to prevent *vimentin* and *snail* upregulation (for both genes *p* < 0.05 vs. CTR) (see Figure 4). Moreover, *vimentin* and *snail* expression confirmed that in all typologies of cells treated with low or high doses, E_2_ was able to accelerate EMT that was already detectable at passage 1 (*p* < 0.001 for both E_2_ and E_2_+P_4_ vs. CTR at passage 1) (see Figure 4). The upregulation effect of E_2_+P_4_ treatment was still evident at passage 3 (*p* < 0.0001 and *p* < 0.001, respectively) when it resulted higher than that in CTR cells (see Figure 4), thus suggesting a marked inductive EMT effect induced by the combination of high doses of steroids.

### 3.4. Steroid Stimulation Influenced Plasticity in Pretreated oAECs

Steroid-pretreated cells were differentiated toward osteogenic or chondrogenic lineage in order to clarify the influence of steroids on oAEC plasticity.

**Figure 5:**
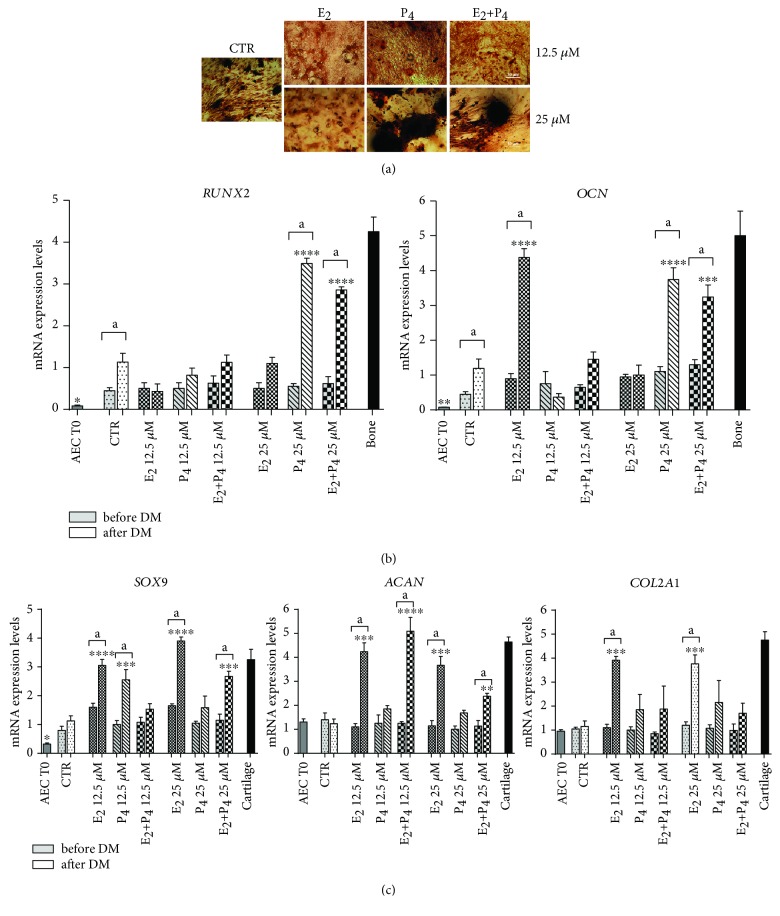
Response of steroids amplified oAEC to osteogenic differentiation. (a) Alizarin red staining was used to evaluate deposition of mineralized matrix nodules in CTR and steroid pretreated oAEC (E_2_, P_4_, or E_2_+P_4_ treatments at 25 *μ*M) after 21 days of culture in osteogenic media (DM). Scale bar = 50 *μ*m. (b) Expression of bone-related genes (*RUNX2* and *OCN*) analysed by real-time qPCR analysis in oAEC after isolation (time 0) and in CTR and steroid pretreated oAEC before (before DM) and after (after DM) osteogenic differentiation. Ovine bone tissue was used as positive control for bone-related genes. (c) Expression of an early (*SOX9*) and two late chondrogenesis-related genes (*ACAN* and *COL2A1*) by real-time qPCR analysis in oAEC after isolation (time 0) and in CTR and steroid pretreated oAEC before (before DM) and after (after DM) osteogenic differentiation. Cartilage tissue was used as positive control for chondrogenesis-related genes. Relative quantification of each mRNA gene expression was calculated using the ΔΔCt method and presented as fold change in gene expression normalized to endogenous *GAPDH* (internal control) and relative to the CTR after DM (calibrator). Data was expressed as mean ± SEM values of samples, each performed in triplicate, obtained at least three different animals. Values statistically different for ^∗^*p* < 0.05, ^∗∗^*p* < 0.01, ^∗∗∗^*p* < 0.001, and ^∗∗∗^*p* < 0.0001 vs. CTR after DM. ^a^Values statistically different for *p* < 0.05 in the same sample before and after DM. CTR: control cell; E_2_: estradiol; P_4_: progesterone.

After 21 days of culture under osteogenic inductive conditions, CTR cells showed extracellular matrix mineralization (see Figure 5(a)) sustained by the upregulation of *RUNX2* and *OCN* expressions (*p* < 0.05 vs. CTR before DM) (see Figure 5(b)). P_4_ supplementation, alone or in combination with E_2_ (E_2_+P_4_), increased cell ability to mineralize the extracellular matrix in a dose-dependent manner (see Figure 5(a)). The high osteogenic ability of P_4_-treated cells was confirmed by *RUNX2* and *OCN* gene expressions that were significantly higher in oAEC pretreated with high dose of P_4_ (25 *μ*M) supplemented alone (*p* < 0.001 vs. CTR after DM) or in combination with E_2_ (*p* < 0.001 vs. CTR after DM) (see Figure 5(b)). On the contrary, E_2_-pretreated oAECs displayed a very limited osteogenic activity either in terms of matrix mineralization or bone-related gene expression that was unaffected by the dosage (see Figures 5(a) and 5(b)). Surprisingly, cells exposed to low doses of E_2_ (12.5 *μ*M) were able to upregulate *OCN* (*p* < 0.001 vs. CTR cells) (see Figure 5(b)) even if in the absence of any extracellular matrix mineralization (see Figure 5(a)). In addition, E_2_-pretreated oAEC displayed, at the end of the osteogenic inductive period, a typical chondro-like morphology characterized by a round shape with a cluster-like organization (see Figure 5(a)). So, in order to confirm this morphological evidence, the cells were also assessed for the expressions of early (*SOX9*) and late (*ACAN* and *COL2A1*) chondrogenesis-related genes (see Figure 5(c)). This analysis confirmed that E_2_-pretreated cells independent of the dosage upregulated early and late chondrogenesis-related genes besides the osteogenic inductive cultural conditions (see Figure 5(c)). E_2_ chondrogenic commitment was partially inhibited by P_4_ supplementation (see Figure 5(c)). In this cell group (E_2_+P_4_), a significant increase in *ACAN* mRNA levels (*p* < 0.05 vs. CTR after DM) was recorded, independent of the dosage (see Figure 5(c)). The early chondrogenic *SOX9* gene was also upregulated in oAEC pretreated with low dosage of P_4_ (see Figure 5(c)).

### 3.5. E_2_ Induces Chondrogenic Differentiation in Pretreated oAEC

**Figure 6:**
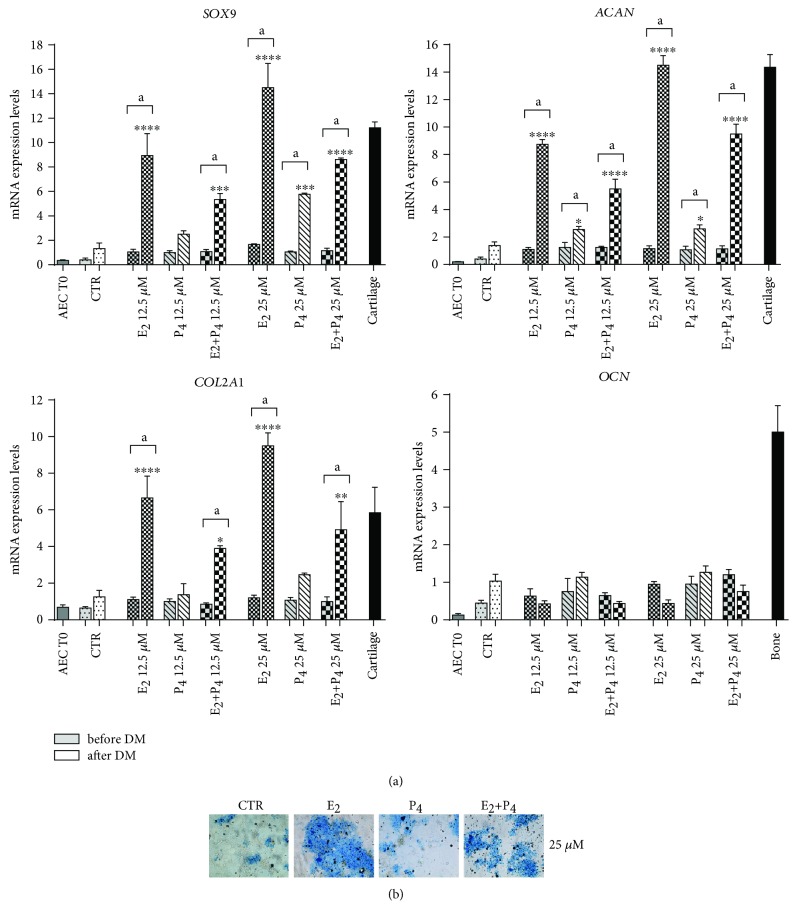
Response of steroids amplified oAEC to chondrogenic inductive cultural conditions. (a) Real-time qPCR analysis of *SOX9*, *COL2A1*, *ACAN*, and *OCN* expression in freshly isolated oAEC (time 0) and in differentiated CTR and steroid pretreated oAEC (E_2_, P_4_, or E_2_+P_4_ at 12.5 *μ*M and 25 *μ*M) after *in vitro* culture in chondrogenic medium for 21 days. Cartilage and bone tissues were used as positive control for gene expression. Relative quantification of each mRNA gene expression was calculated using the ΔΔCt method and presented as fold change in gene expression normalized to endogenous *GAPDH* (internal control) and relative to the CTR after DM (calibrator). Data was expressed as mean ± SEM values of samples, each performed in triplicate, obtained at least three different animals. Values statistically different for ^∗^*p* < 0.05, ^∗∗^*p* < 0.01, ^∗∗∗^*p* < 0.001, and ^∗∗∗^*p* < 0.0001 vs. CTR after DM. ^a^Values statistically different for *p* < 0.05 in the same sample before and after DM. (b) Representative images of Alcian blue staining to assess deposition of proteoglycans in the extracellular matrix in CTR and steroid pretreated oAEC (E_2_, P_4_, or E_2_+P_4_ at 25 *μ*M) after chondrogenic differentiation. Scale bar = 50 *μ*m. CTR: control cell; E_2_: estradiol; P_4_: progesterone.

Analogously, the effect of steroids was verified on oAEC chondrogenic plasticity by assessing gene expression profiles of early and late chondrogenesis-related (*SOX9*, *ACAN*, and *COL2A1*) as well as proteoglycan deposition (see Figure 6(b)). The late osteogenic (*OCN*) genes were also analysed (see Figure 6(a)). The incubation carried out in standardized chondrogenic inductive conditions promoted a slight differentiation in oAEC amplified in the absence (CTR) or in presence of P_4_ as indicated by the Alcian blue positivity (see Figure 6(a)). and by the low expression of chondrogenic genes (see Figure 6(b)). P_4_-treated cells at high dosage, despite the slight degree of proteoglycans deposition, displayed only a significant upregulation of early chondrogenic gene, *SOX9* (*p* < 0.001 vs. CTR after DM) (see Figure 6(a)). The chondrogenesis significantly improved in oAEC amplified with E_2_ even if differentiation resulted strictly dose-dependent (see Figure 6). In particular, oAEC amplified with high doses of E_2_ displayed a dramatic upregulation of *SOX9*, *ACAN*, and *COL2A1* (*p* < 0.0001 vs. CTR after DM) (see Figure 6(a)) combined with a massive deposition of proteoglycans in extracellular matrix (see Figure 6(b)). This chondrogenic inductive effect promoted by E_2_ was always counteracted in cells amplified with the simultaneous presence of P_4_ (see Figures 6(a) and 6(b)). In this cell group, the expression of chondrogenesis-related genes was significantly lower than that recorded in E_2_-treated ones but it was always higher than that of CTR (see Figure 6(a)). The *OCN* gene expression was unaffected (see Figure 6(a)) independently of the amplification conditions considered.

## 4. Discussion

The present study demonstrated that E_2_ and P_4_ could be supplemented during the oAEC amplification protocols in order to modulate their properties according to therapeutic use.

In more detail, steroid supplementation may be adopted to control oAEC stemness, phenotype, and mesenchymal plasticity. As previously proposed [30], P_4_ (25 *μ*M) addition to cultural media could be a useful strategy to preserve stemness gene expression that it is rapidly depleted during amplification [30] under CTR or E_2_ conditions_,_ without affecting cell proliferation except at the beginning of the culture. In addition, long-term P_4_ exposure supported the preservation of oAEC epithelial phenotype by determining a clear precommitment of the cells towards the osteogenic differentiation lineage. On the contrary, E_2_ addition rapidly induced EMT by facilitating oAEC in undertaking the chondrogenic differentiation and switching off the osteogenic one. Both these evidences seem to support the idea that steroid supplementation may be gainfully employed to develop tissue-targeted stem cell-based therapy.

These valuable results have been obtained by treating oAEC with high concentrations of steroids apparently far from the physiological dosage [44, 45]. Unfortunately, it is hardly complex to determine *in vitro* the steroids cell availability in the absence of the transport mechanisms regulating lipid hormone balance physiologically. This may explain why the steroid modulatory effects became evident at concentration higher than *μ*M [30, 36–38, 44–47] instead of the lower ones driving pregnancy and the luteal reproductive cycle [44, 45]. Independent of the physiological or pharmacological meaning of steroid supplementation used, both E_2_ and P_4_ during oAEC amplification showed a clear dose- and steroid-dependent influence.

Immediately after isolation, oAEC expressed a multilineage differentiation ability [4] despite their epithelial phenotype [4, 14, 30]. The large plasticity of AEC has attracted increasing attention to propose them as a valid and a more safe alternative to embryonic stem cells [9, 24, 48–59]. Nevertheless, in order to move AEC towards translation to clinical practice, there are still knowledge gaps that remain to be investigated.

Recently, several groups have clearly pointed out the needs to adopt new quality assessment technologies for protocols of isolation, expansion, and differentiation in order to determine the exact AEC status before transplantation to better orient their use towards the treatment of specific diseases [28, 30].

It is clear now that oAEC expansion may affect cell phenotype and biological properties [14, 30] in different models [28–30]. In particular, AECs spontaneously undergo epithelial-mesenchymal transition (EMT) [28–30] during expansion, a transdifferentiation process whereby epithelial cells acquire a mesenchymal phenotype with a wider migratory and invasive properties. EMT may play a role in AEC healing properties [31] even if this process needs to be controlled *in vitro* to have clear information of the regenerative potential before proceeding to cell transplantation. EMT is basically switched on *in vitro* though the activation of paracrine/autocrine signals TGF-*β*-mediated [28, 53]. This growth factor is an EMT-regulating signaling linked to the family of transcription factors (EMT-TFs) leading to the loss of epithelial proteins (i.e., E-cadherin and cytokeratin-8) and the upregulation of mesenchymal determinants such as vimentin and *α*-SMA [54, 55]. P_4_ supplementation during oAEC amplification inhibited the *in vitro* TGF-*β* paracrine/autocrine loop and the relative intracellular signaling thus maintaining the native epithelial phenotype [30]. Relevantly, the evidence that the long-term preservation of epithelial phenotype P_4_ induced was even positively correlated with the persistence of oAEC immunomodulatory activity and with the cell ability in releasing higher levels of anti-inflammatory cytokines under basal and stimulating conditions [30]. With the present research, a strong precommitment towards osteogenic lineage has been also associated to P_4_ supplementation. This latter evidence further clarified the functional impact of this novel amplification protocols by adding new evidence on the effects of steroids [34, 35] that was previously recognized to be involved in modulating EMT also in other cell typologies [38, 39] and also in metastatic breast cancer cells [36, 37, 57].

In addition, the opposite E_2_ effects during oAEC amplification have been confirmed. Indeed, high dosage of E_2_ (25 *μ*M), alone or in combination with P_4_, was able to accelerate the process of EMT by increasing the incidence of *α*-SMA protein and upregulating the EMT-related genes (*vimentin* and *snail* gene expression levels) as previously demonstrated, analogously in cancer cell models [57–59] and in hESCs [60]. However, different from hESCs [60] and oAEC amplified under P_4_ and P_4_+E_2_ long-term conditions, E_2_ exposure induced a downregulation in pluripotent genes in oAEC. The practical impact of this has to be studied more in details since it may strongly influence stem cell plasticity. Surely, cell expanded with E_2_ became more sensitive to chondrogenesis that can be only weakly induced in amniotic-derived cells amplified under CTR or P_4_ conditions using standardized *in vitro* chondroinductive protocols. On the contrary, oAEC amplified with P_4_ was easily committed towards the osteogenic lineage. Interestingly, it was noticed that E_2_ and P_4_ pretreatments were able to give priority to differentiation towards a specific mesenchyme tissue lineage, P_4_ for osteogenic and E_2_ for chondrogenic, by switching off at the same time either of the other differentiation signal. This is quite clear during oAEC exposure to E_2_: treated cells not only did not display any osteogenic plasticity but also, on the contrary, converted the osteogenic inductive stimulus into a chondrogenic one.

In order to partially counteract the powerful mesenchymal effects of E_2_, oAEC amplification can be performed in combination with P_4_. Probably, P_4_ and E_2_ interacted on the closely intertwined pathways controlling chondrogenesis and osteogenesis by providing a targeted tune on the related intracellular signals involved [61]. Some speculation on these regulatory mechanisms involved in oAEC steroid precommitment could be advanced even if they were not investigated in detail yet. For example, the specific mesenchymal attitude verified in E_2_-treated AEC may be ascribable to the chondrogenic inductive role of TGF-*β* [61, 62] that usually increases in culture when cells have experienced EMT [28, 30]. The effects of TGF-*β* superfamily on chondrogenisis were transduced by SMAD family members that, in turn, were able to regulate in cooperation with *SOX9*, the expression of gene *COL2A1*, a terminal molecule of the process of cartilage development and regeneration [61]. On the contrary, TGF-*β* appears to play as an osteogenic inhibitor at least when added to high-density culture of periosteum-derived cells [63]. Surely, the cultural levels of TGF-*β* significantly decrease when P_4_ is added during oAEC amplification [30].

Altogether, these findings may impact the oAEC use in regenerative medicine and, in particular, may orient the development of novel cell-based strategies for repairing musculoskeletal defects.

In our hands, oAECs have been already tested for their bone- and tendon-related regenerative capacity under allo- and xenotransplantation procedures [17, 20–23, 64]. In both these experimental models, hAEC and oAECs displayed a powerful regenerative activity exerted either by potentiating the endogenous recruitment of endogenous progenitor cells through positive paracrine mechanisms or by direct contributing to tissue-specific healing through an *in situ* transdifferentiation [9]. On the contrary, sporadic researches linked AEC to *in vivo* chondrogenesis to date a part from a paper that proposed to repair a full-thickness femoral cartilage defects in sheep using the *in toto* amniotic membrane [65].

In this specific therapeutic context, steroid treatments could represent an innovative tool aimed at precommitting AEC before transplantation in bone and/or cartilage defects. In this context, P_4_ may potentially be useful to improve the AEC bone regenerative action enabling the transplanted cells [30]. On the other hand, E_2_ treatments may offer new solution to overcome the clinical challenges of cartilage disorders. The availability of both osteo- and chondro-oriented cells could be proposed to tempt tissue-oriented/tissue-engineered graft or patch solutions to face the still-difficult regeneration of osteochondral defects [66]. The osteochondral repairing requires, indeed, to switch on *in situ* different mechanisms able to support, operating in a synergic manner, bone and cartilage interfaces. The tissue engineering solutions could take advantage from the developed technology of biphasic scaffolds mimicking the specificity of bone and chondrotissue microarchitecture by combining it with the use of *in vitro* steroid precommitment AEC.

## 5. Conclusions

In conclusion, the present results demonstrated for the first time that prolonged steroid treatments can modify oAEC biological properties and plasticity. Steroid treatments may be proposed as innovative *in vitro* strategy to induce oAEC precommitment, opening new prospective for their use in stem cell-based therapy addressed to cure bone and/or cartilage defects.

## Figures and Tables

**Table 1:** Sequences of primers and conditions used in real-time qPCR.

Gene	Accession no.	Primer sequences	Cycles	Annealing Tm	Efficiency (*E*)
*Vimentin*	XM_004014247.3	F: 5′-GACCAGCTCACCAACGACA-3′	40	65.2	1.94
	Ovine	R: 5′-CTCCTCCTGCAACTTCTCCC-3′			
*Snail 1*	XM_004014881.2	F: 5′-GTCGTGGGTGGAGAGCTTTG-3′	40	66.4	1.96
	Ovine	R: 5′-TGCTGGAAAGTGAGCTCTGG-3′			
*OCN*	DQ418490.1	F: 5′-AGACACCATGAGAACCCCCAT-3′	40	61	1.95
	Ovine	R: 5′-TTGAGCTCACACACCTCCCT-3′			
*RUNX2*	Multiple alignment [7]	F: 5′-GGACGAGGCAAGAGTTTCAC-3′	40	66	1.91
		R: 5′-GGTGGCAGTGTCATCATCTG-3′			
*SOX9*	XM_015098410.1	F: 5′-AGGCTCGAACACGTTCCCC-3′	40	61.12	1.97
	Ovine	R: 5′-GTTCAGCAGTCTCCAGAGCTT-3′			
*COL2A1*	XM_012174384.2	F: 5′-ACCAGGACCAAAGGGACAGA-3′	40	60.25	1.99
	Ovine	R: 5′-AAATCCACCAGCCATCTGGG-3′			
*ACAN*	XM_012098454.2	F: 5′-AGTCAGTGGTGACTTCACAGG-3′	40	60.1	1.94
	Ovine	R: 5′-GGCAACCTGTCAACTATGGG-3′			
*SOX2*	X96997.1	F: 5′-CACCCGCATGTACAACATGAT-3′	45	67.7	1.92
	Ovine	R: 5′-TCTTAGGATTCTCTTGGGCCA-3′			
*OCT4*	NM_174580.1	F: 5′-CTGCAGAAGTGGGTGGAGGAA-3′	45	68.7	1.94
	Bovine	R: 5′-CTGCAGTGTGGGTTTCGGGCA-3′			
*NANOG*	FJ970651.1	F: 5′-TGGATCTGCTTATTCAGGACAG-3′	45	65.4	1.96
	Ovine	R: 5′-TGCTGGAGGCTGAGGTATTTC-3′			
*GAPDH*	AF030943.1	F: 5′-TCGGAGTGAACGGATTTGGC-3′	40	64.4	1.99
	Ovine	R: 5′-CCGTTCTCTGCCTTGACTGT-3′			

## Data Availability

The data used to support the findings of this study are available from the corresponding author upon reasonable request.
